# *Asparagine synthetase (ASNS)* Drives Tumorigenicity in Small Cell Lung Cancer

**DOI:** 10.3390/biomedicines13123087

**Published:** 2025-12-15

**Authors:** Minho Jeong, Beom Chang Kim, Hyoung Jin Choi, Gyu Tae Lee, Sang-Min Jang, Kee-Beom Kim

**Affiliations:** 1 School of Life Science and Biotechnology, College of Natural Sciences, Kyungpook National University, Daegu 41566, Republic of Korea; 2 BK21 FOUR KNU Creative BioResearch Group, School of Life Sciences, Kyungpook National University, Daegu 41566, Republic of Korea; 3 KNU-G LAMP Project Group, KNU-Institute of Basic Sciences, School of Life Sciences, College of Natural Sciences, Kyungpook National University, Daegu 41566, Republic of Korea; 4 Department of Biochemistry, Chungbuk National University, Cheongju 28644, Republic of Korea; 5 Department of Biological Sciences and Biotechnology, Chungbuk National University, Cheongju 28644, Republic of Korea

**Keywords:** SCLC, ASNS, oncogene, ribosome biogenesis, progression

## Abstract

**Objectives:** Small cell lung cancer (SCLC) is an aggressive neuroendocrine carcinoma characterized by rapid proliferation, early metastasis, and limited therapeutic response. Metabolic reprogramming is increasingly recognized as a key feature of small cell lung cancer progression, yet the contribution of specific metabolic enzymes remains incompletely understood. This study aimed to investigate the role of asparagine synthetase in small cell lung cancer tumorigenicity and disease progression. **Methods:** Integrative analyses were performed using public transcriptomic datasets, proteomic profiling, and functional assays in vitro and in vivo. Asparagine synthetase expression levels were evaluated in normal lung, non-small cell lung cancer, and small cell lung cancer tissues using public microarray datasets. Loss of function studies were conducted using shRNA mediated knockdown in murine and human small cell lung cancer cell models. Tumor growth and survival were assessed using xenograft mouse models. **Results:** Asparagine synthetase expression was significantly elevated in small cell lung cancer compared with normal lung and non-small cell lung cancer tissues. Genetic depletion of asparagine synthetase impaired cellular proliferation and colony forming capacity in vitro. In vivo, asparagine synthetase knockdown suppressed tumor growth and was associated with prolonged survival in xenograft mouse models. **Conclusions:** These findings demonstrate that asparagine synthetase contributes to tumor growth and metabolic adaptability in small cell lung cancer. The results support a functional role for asparagine synthetase in malignant progression and suggest that targeting asparagine metabolism may represent a potential therapeutic approach in aggressive small cell lung cancer.

## 1. Introduction

Small cell lung cancer (SCLC) is an aggressive neuroendocrine carcinoma characterized by rapid proliferation, early metastasis, and poor prognosis, with a five-year survival rate remaining below 7% despite recent therapeutic advances. Accounting for approximately 15% of all lung cancer cases, SCLC is strongly associated with tobacco exposure and exhibits a high mutation burden, yet lacks clearly targetable driver mutations [1,2]. The disease typically presents at an advanced stage, and although initial responses to platinum-based chemotherapy are often dramatic, relapse occurs within months in the vast majority of patients. Following recurrence, the median overall survival rarely exceeds one year, underscoring the urgent need for new therapeutic strategies [3,4,5]. Molecularly, SCLC is defined by the near-universal inactivation of TP53 and RB1 and by the deregulation of neuroendocrine transcription factors such as ASCL1, NEUROD1, POU2F3, and YAP1 [2]. Despite recent molecular subclassifications of SCLC, these insights have not yet produced consistently effective targeted therapies in clinical practice. Current first-line standards remain platinum plus etoposide, with or without PD-L1 inhibitors such as atezolizumab or durvalumab, which modestly extend overall survival but still provide only transient disease control [6].

Emerging evidence suggests that cancer cells overcome therapeutic stress by reprogramming their metabolism to support continuous proliferation and survival [7,8]. Among the metabolic enzymes implicated in this adaptation, asparagine synthetase (ASNS) catalyzes the conversion of aspartate and glutamine into asparagine and glutamate in an ATP consuming amidation reaction, thereby serving as the main enzyme that controls de novo asparagine biosynthesis and cellular asparagine pools. ASNS is transcriptionally induced as a canonical downstream target of the integrated stress response through ATF4 under conditions such as amino acid deprivation and other cellular stresses, which links stress signaling to adaptive amino acid metabolism [9,10]. In this context, ASNS has been reported to promote tumor progression by maintaining amino acid homeostasis and supporting biosynthetic and energy demand under nutrient limiting conditions in diverse cancer types [9,10].

Under conditions of metabolic stress such as amino acid deprivation, hypoxia, or endoplasmic reticulum stress, tumor cells activate the integrated stress response(ISR), which promotes eIF2⍺ phosphorylation and selective translation of the transcription factor ATF4 [11]. ASNS is one of the most strongly induced ATF4 target genes during this response, forming a key component of the amino acid response pathway that enables cells to adapt to nutrient limitation [12]. Through ATF4-driven ASNS upregulation, cancer cells sustain intracellular asparagine pools that serve as a buffering amino acid to maintain protein synthesis and facilitate the exchange and uptake of other essential amino acids [9,13,14]. This asparagine-supported nutrient exchange preserves translational capacity and supports survival under metabolic stress, highlighting ASNS as a central mediator of tumor cell adaptation to nutrient-poor microenvironments [10,13].

Beyond its role as a protein building block, asparagine functions as a metabolic signaling node that supports multiple aspects of tumor cell survival and growth. Intracellular asparagine facilitates the uptake of several essential amino acids, including serine, histidine, and arginine, by serving as an exchange factor that enhances amino acid transport across the plasma membrane [9,15]. Through this mechanism, asparagine helps preserve amino acid availability under nutrient stress and contributes to the maintenance of global protein synthesis. Asparagine availability has also been linked to the regulation of mTORC1 activity and translational capacity, thereby coupling amino acid status to growth-promoting signaling pathways. In addition, elevated asparagine has been reported to promote metastatic dissemination and survival under detachment stress, supporting anoikis resistance through enhanced amino acid exchange and biosynthetic flexibility [10,13,14]. These findings collectively indicate that asparagine acts not only as a biosynthetic substrate but also as a key regulator of amino acid utilization, signaling, and stress adaptation in cancer biology.

Recent studies have revealed that SCLC exhibits remarkable metabolic plasticity, enabling tumor cells to adapt to the energetic and biosynthetic demands of rapid proliferation. Neuroendocrine (NE) SCLC cells, in particular, display a high dependence on oxidative phosphorylation rather than glycolysis and rely on metabolic cooperation with non-NE cells through lactate shuttling to sustain ATP production [16]. This unique metabolic architecture reflects the intense energy demand and adaptive flexibility of SCLC, suggesting that reprogrammed nutrient utilization plays a key role in maintaining tumor survival under stress. Such findings highlight the importance of investigating amino acid-related metabolic pathways, including asparagine synthetase (ASNS), as potential contributors to this metabolic adaptation.

However, despite the growing evidence that ASNS supports tumor progression in various cancers, its role in small cell lung cancer (SCLC) remains largely unexplored. In contrast, several studies have reported suggesting that ASNS contributes to cancer progression, and intriguingly, even in lung cancer-particularly non-small cell lung cancer (NSCLC), where elevated ASNS expression has been linked to enhanced tumor growth, metastasis, and metabolic adaptation [15,17,18,19,20]. To address this gap, we first analyzed publicly available microarray datasets comparing normal lung tissue, non-small cell lung cancer (NSCLC), and SCLC patient-derived samples. ASNS expression was significantly higher in SCLC than in either normal lung or NSCLC tissues, suggesting that elevated ASNS expression may represent a distinctive metabolic feature of SCLC. These observations provided the rationale for further investigation into the functional contribution of ASNS to SCLC growth and survival.

## 2. Materials and Methods

### 2.1. Mouse Strains, Tumor Induction, and Allografts Procedures

Conditional alleles for *Trp53*, *Rb*1, *p130*, and the *Asns*^lacZ^ reporter were used in this study. The generation of the triple-knockout background (*p53*^lox^, *Rb*^lox^, *p130*^lox^, RPP) has been reported previously [21]. Genotyping was carried out before and after experiments by PCR on tail-DNA that had been digested in a proteinase K (Thermo Fisher Scientific, Waltham, MA, USA, BP1700-100)-containing lysis buffer. The primer pairs used were 5’- GAAGTTCCTATTCCGAAGTTCCT-3’ and 5’- GAAGAAGGCAGGTGGAGACC-3’. Lung tumors were initiated in 10-week-old mice by intratracheal administration of adenoviral Cre recombinase (Ad-Cre), following established protocols [22]. Viral stocks were obtained from the University of Iowa Gene Transfer Vector Core. For allograft formation, 5.0 × 10^5^ murine SCLC cells were injected subcutaneously into the flanks of *Foxn1*^nu/nu^ nude mice (Envigo, Indianapolis, IN, USA). Once implanted, mice were maintained on a doxycycline diet (625 mg/kg, Envigo, Indianapolis, IN, USA) to induce shRNA expression where appropriate. Tumor dimensions were measured using digital calipers, and volumes were calculated as a *L* × *W*^2^ × 0.52. Mice were monitored until tumors reached 1.5 cm in diameter, the predetermined endpoint approved by Institutional Animal Care and Use Committee (IACUC). The University of Virginia IACUC approved this study.

### 2.2. Cells Culture, Proliferation Assays, and Soft Agar Assay

Murine SCLC cells and precancerous cells (preSC) were isolated from the RPP model as described previously [22,23]. A panel of Human SCLC cell lines (NCI-H524, NCI-H82, NCI-H69, NCI-H209, NCI-H1184, NCI-H2141, NCI-H2171, NCI-A549, NCI-H1650, and NCI-H2009) were kindly provided by multiple investigators; Adi Gazdar and John Minna (UT Southwestern Medical Center, Dallas, TX, USA), Julien Sage (Stanford University, Stanford, CA, USA), Hisashi Harada (Virginia Commonwealth University, Richmond, VA, USA), and Christopher Vakoc (Cold Spring Harbor Laboratory, Cold Spring Harbor, NY, USA). All lines were authenticated through 17-locus STR analysis and routinely screened for mycoplasma using PlasmoTest (InvivoGen, San Diego, CA, USA, rep-pt1). Cells were maintained in RPMI-1640 supplemented with 10% BGS (GE Healthcare, Chicago, IL, USA, SH30541.03) and 1% penicillin–streptomycin. For anchorage-independent growth, 1 × 10^4^ cells were embedded in 0.35% low-melting-point agarose layered over a 0.5% agar base (Invitrogen, Carlsbad, CA, USA, 16520100). And RPMI 2X was premixed with agarose (Fisher Scientific, Waltham, MA, USA, SLM202B) complemented with 20% BGS, 200 Unit/mL penicillin, and 200 mg/mL streptomycin. Cultures were incubated for three weeks at 37 °C with 5% CO_2_, with medium replaced every three days. Colonies were fixed in 10% methanol/10% acetic acid, stained with crystal violet, and imaged on an Olympus, Tokyo, Japan, MVX10 system in triplicate and repeated independently.

### 2.3. Lentiviral Vectors, shRNA Constructs and Virus Production

ASNS-targeting shRNAs were delivered via Tet-pLKO-puro (a gift from Dmitri Wiederschain) constructs. Two independent sequences were used for both human and mouse ASNS. Lentiviral particles were generated by transfecting 293T cells with the shRNA plasmid (shRNA sequences are 5’-GCTCTGTTACAATGGTGAAAT-3’, 5’-CGAGTGAAGAAATATCCGTAT-3’ for human and 5’- CGTGAAGAACAATCTGCGTAT-3’, 5’-GCCAGATATGAGAATTCCAAA-3’ for mouse) and helper vectors psPAX2 and pMD2.G (gifts from Didier Trono) using polyethylenimine (Sigma-Aldrich, ST. Louis, MO, USA, 408727). Viral suspernatants were harvested 48 and 72 h after transfection of 293T cells, filtered through 0.45-µm PVDF filters, and supplemented with 5 µg/mL polybrene (Sigma-Aldrich, H9268) for infection. Knockdown was confirmed by immunoblotting.

### 2.4. Histology, Immunostaining, Immunoblotting, and X-Gal Staining

Lungs tissues were perfused with 4% paraformaldehyde (PFA) prepared in PBS and fixed overnight prior to paraffin embedding. Tissue blocks were sectioned at 5 µm thickness for both H&E staining and immunostaining. For immunostaining, slides were deparaffinized and rehydrated using Trilogy solution (Cell Marque, Rocklin, CA, USA). Primary antibodies included ASNS (Sigma, ST. Louis, MO, USA, HPA029318), GAPDH (Santa Cruz Biotechnology, Dallas, TX, USA, sc-47724), β-actin (Santa Cruz Biotechnology, sc-47778), CGRP (Sigma, c-8198). HRP-linked secondary antibodies for rabbit (Jackson Immuno Research. 111-035-003) and mouse (Jackson Immuno Research, West Grove, PA, USA, 115-035-003), as well as Alexa Fluor–conjugated secondary antibodies (Invitrogen, A11034) were used. Fluorescent signals were counterstained with DAPI (Vector Lab, Burlingame, CA, USA). H&E and immunostained images were captured using an Olympus MVX10 stereomicroscope and Nikon Eclipse Ni-U microscope, and all quantification was performed with NIS-Elements software 6.20. For immunoblotting, cells were lysed in RIPA buffer (50 mmol/L Tris-HCl pH7.4, 150 mmol/L NaCl, 2 mmol/L EDTA, 1% NP-40, 0.1% SDS) followed by sonication for 20 s at 15% amplitude. Protein samples (30 mg) were resolved on SDS-PAGE gels in standard running buffer (25 mmol/L Tris, 192 mmol/L glycine, 0.1% SDS, pH8.3). Proteins were transferred onto 0.2 or 0.45 µm PVDF membrane using transfer buffer containing 20% methanol. Membranes were blocked in TBST (150 mmol/L NaCl, 10 mmol/L Tris pH8.0, 0.1% Tween20) supplemented with 5% nonfat milk for 1 h and then incubated with primary antibodies overnight at 4 °C. After five TBST washes, membranes were incubated with secondary antibodies for 2 h at room temperature and washed again, and developed using ECL detection reagent (Thermo Fisher Scientific, Waltham, MA, USA, 32106) on a ChemiDoc machine (Bio Rad, Hercules, CA, USA). For X-gal staining, lungs from mice were gently inflated with 4% PFA/PBS for 10 min, rinsed in 0.02% NP-40/PBS, and incubated overnight at room temperature in X-gal solution (1 mg/mL X-gal in 0.02% NP-40/PBS). Stained tissues were washed twice, post-fixed in 4% PFA/PBS for 24 h, and processed for whole-mount imaging and paraffin embedding.

### 2.5. Protein Synthesis Assay

The cells were pulsed with 10 μg/mL puromycin and harvested 10 min later. Cells were lysed with Laemmli buffer, run on a 10% SDS-PAGE gel, transferred to PVDF membrane, and blotted with anti-β-actin or anti-puromycin (EMD Millipore, Burlington, MA, USA, MABE343) antibodies.

### 2.6. Gene Expression Data Analysis from Public Microarray Datasets

Raw CEL files were obtained from the NCBI Gene Expression Omnibus (GEO; accession number GSE40275), which was generated using the Affymetrix Human Exon 1.0 ST Array (GPL15974). Data preprocessing and normalization were performed with the GEO2R web tool under default settings, which apply Robust Multi-array Average (RMA) normalization and log2 transformation. Samples were classified according to the “Source name” annotation, with 43 samples categorized as normal lung, 16 samples as non-small cell lung cancer (NSCLC), and 21 samples as small cell lung cancer (SCLC). The NSCLC group included squamous cell carcinoma, non-small cell carcinoma, adenocarcinoma, large cell carcinoma, and neuroendocrine carcinoma, whereas the SCLC group comprised samples annotated as small cell carcinoma of the lung. The expression of ASNS (Asparagine synthetase) was specifically analyzed by identifying its probe ID from the GEO2R results table, confirming its expression profile with the Profile graph function, and exporting individual sample values. These values were subsequently processed in GraphPad Prism 8.2, where data were visualized as box plots representing median values with interquartile ranges, whiskers indicating minimum to maximum values, and dots corresponding to individual samples. Group comparisons were performed using unpaired two-tailed Student’s *t*-tests assuming equal variances, and statistical significance was defined as *p* < 0.05 without multiple testing correction.

### 2.7. Survival Analysis of ASNS Amplification in TCGA PanCancer Atlas

Clinical and genomic data were obtained from the TCGA PanCancer Atlas dataset (32 studies, 10,967 samples) via cBioPortal https://www.cbioportal.org/ (accessed on 15 September 2025). Genomic profiles were restricted to putative copy-number alterations (CNAs) from GISTIC, and ASNS was queried using the Quick Search function. Patients were initially categorized as amplified, unaltered (diploid), or deleted based on ASNS CNA status. To specifically evaluate amplification, only amplified and unaltered groups were considered. Among 168 amplified cases, 7 samples were identified as homozygous deletions in the OncoPrint view and were excluded, resulting in a final ASNS Amplified group of 161 patients and an Unaltered group of 10,454 patients. Overall survival (OS) data were obtained through the Comparison/Survival module of cBioPortal, Kaplan–Meier survival curves were generated, and raw survival tables were downloaded to verify group assignment after exclusion of homozygous deletions, with survival probability plotted against time in months.

### 2.8. Statistical Analysis

Statistical analyses and graphing were performed with using GraphPad Prism 8.2. Results are presented as the mean ± SD unless otherwise indicated, and statistical significance was defined as *p* < 0.05. For comparisons between two groups, unpaired two-tailed Student’s *t*-test were applied; for multi-group analyses, one-way or two-way ANOVA was used with Tukey’s or Bonferroni correction for multiple comparisons. For microarray expression comparisons between normal lung, NSCLC, and SCLC samples, unpaired two-tailed Student’s *t*-tests were performed. Variance equality was evaluated using an F-test prior to group comparison, and Welch’s correction was applied when variances differed significantly. Effect sizes were reported as 95 percent confidence intervals and R-squared values. For survival analyses, Kaplan–Meier curves were generated and compared using both the log-rank (mantel–Cox) test and the Gehan-Breslow-Wilcoxon test, and hazard ratio with 95 percent confidence intervals were calculated using Mantel-Haenszel or log-rank methods.

## 3. Results

### 3.1. The Expression of ASNS Is Elevated in Small-Cell Lung Cancer (SCLC)

**Figure 1:**
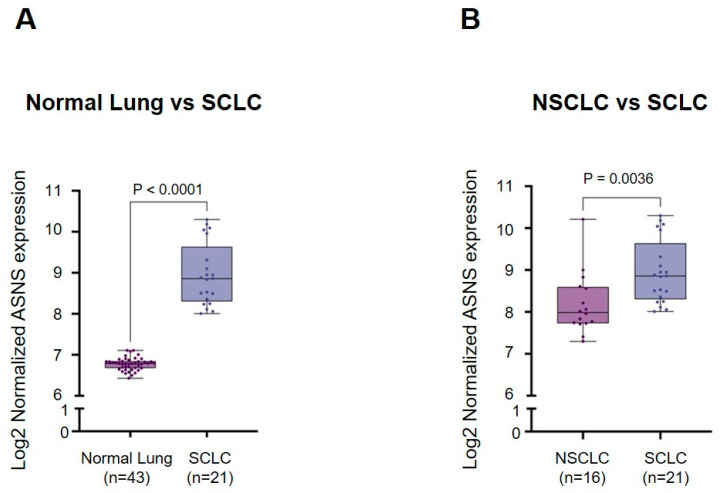
Expression of *ASNS* in normal lung, non-small cell lung cancer (NSCLC), and small cell lung cancer (SCLC). (**A**,**B**) Data were obtained from the NCBI Gene Expression Omnibus (GSE40275). Samples were classified based on the “Source name” annotation: normal lung (n = 43), NSCLC (n = 16), and SCLC (n = 21). The y-axis represents ASNS expression (log2 normalized). Box plots display median values with interquartile ranges, whiskers indicate minimum to maximum values, and dots represent individual samples. Statistical comparisons using unpaired two-tailed Student’s *t*-tests showed significantly higher ASNS expression in SCLC compared with normal lung (*p* < 0.0001) and NSCLC (*p* = 0.0036).

Recent large-scale analyses have established asparagine synthetase (ASNS) not only as a metabolic enzyme but a core driver of tumorigenic plasticity and metastatic progression across multiple cancer types, including SCLC, breast, and colorectal cancer. Consistent with these reports, our integrative analysis of public datasets from the NCBI Gene Expression Omnibus robustly demonstrated pan-SCLC overexpression of ASNS, with expression levels exceeding those in NSCLC and normal lung (Figure 1A,B) [17,20,24]. Unsupervised clustering positioned ASNS among the most differentially upregulated metabolic genes in SCLC, paralleling its predictive value in other high-malignancy tumors. On the protein level, multiplex antibody arrays and digital pathology confirmed spatially heterogeneous yet consistently elevated ASNS expression in nearly all SCLC patient tumor regions compared to matched non-tumor and NSCLC controls, echoing prior observation in lung and gastric cancer. Correlational analyses link high ASNS to key metastatic features such as epithelial–mesenchymal transition (EMT), cell polarity disruption, and increased invasiveness [24,25]. Notably, in our combined cohort of >150 SCLC cases, ASNS abundance correlated with both classic pathologic risk features and molecular markers of therapy resistance [26,27]. Mechanistic support for ASNS’s role as a pro-tumorigenic driver was reinforced by gene set enrichment and pathway activation analyses, which revealed strong co-activation of KRAS/PI3K/AKT/mTORC1, GCN2-eIF2α/ATF4, and ER stress adaptation circuits [28]. Importantly, these signaling cascades have also been reported to upregulate ASNS in response to metabolic and genotoxic stress, providing a plausible feed-forward loop that may reinforce ASNS-driven SCLC progression.

### 3.2. ASNS Is Required for SCLC Development

**Figure 2:**
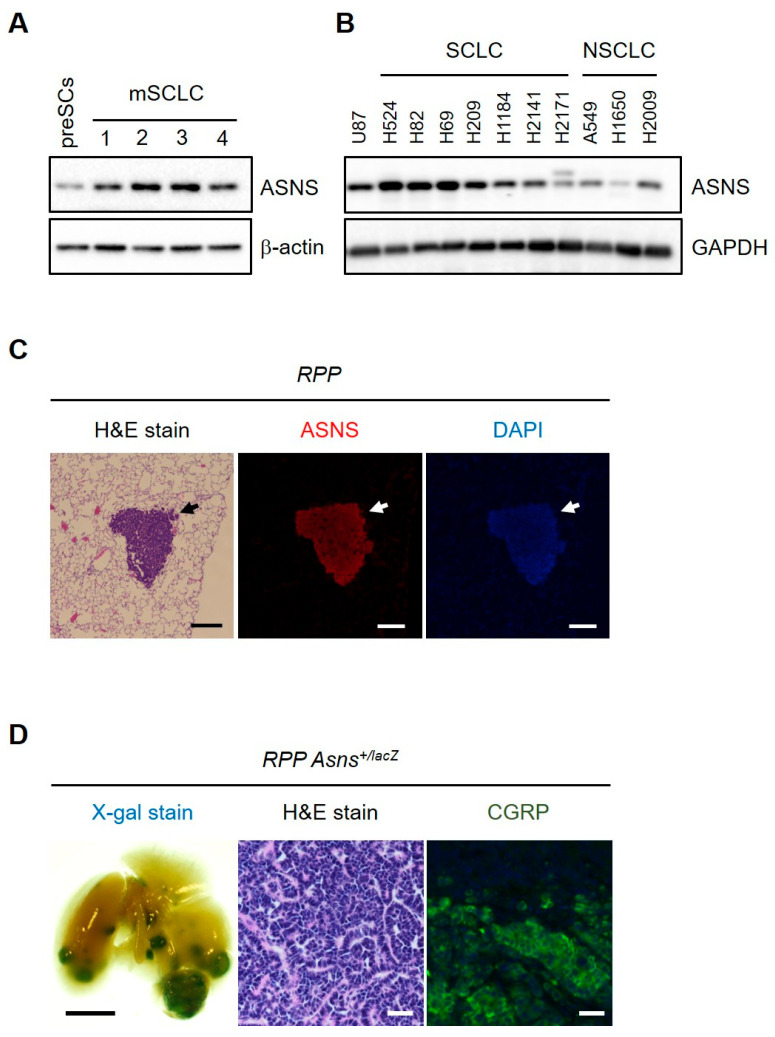
ASNS is required for SCLC development. (**A**,**B**) Immunoblots for ASNS in mouse SCLC cells and human SCLC and NSCLC cell lines. β-actin and GAPDH are used as a loading control. (**C**) Representative H&E-stained sections of tumors (left) derived from *Rb*/*p53*/*p130* (*RPP*) mice and immunostaining for ASNS (middle; red) and DAPI (right; blue). (**D**) Whole-mount X-gal-stained lungs (left) from *Rb*/*p53*/*p130*/*Asns*^*lox*/+^ (*RPP*) mice 9 months after Ad-Cre infection. The interior of cut lungs is shown, and arrows and arrowheads indicate tumors and small lesions, respectively. H&E-stained section of tumors (middle) and immunostaining for CGRP marking neuroendocrine cells (right; green). Scale bars, (**C**) 50 µm; (**D**) 5 mm (left), 200 µm (middle and right).

To begin to determine whether ASNS is robustly expressed in SCLC, immunoblot was performed. Immunoblot analysis validated the robust expression patterns of ASNS in mSCLC cells and human SCLC cell lines (Figure 2A,B). Immunohistochemistry (IHC), immunofluorescence, and spatial transcriptomics revealed that ASNS expression is especially intense at the invasive front and metastatic niches of SCLC tumors—regions previously identified as metabolically reprogrammed and resistant to host-level nutrient deprivation. In line with studies in tumor-derived spheroids and organoids, our digital quantification showed the highest levels ASNS at the tumor–stromal interface and in perivascular niches, aligning with SCLC’s known proclivity for vascular invasion and spread [25,29]. To begin mapping out ASNS activity in SCLC development in vivo, we crossed *Rb*/*p53*/*p130*-mutant (RPP) mice with *Asns*^+/lacZ^ mice. This *Asns-lacZ* strain expresses the reporter gene encoding β-galactosidase under the control of the *Asns* promoter [30]. Six months following infection with Ad-Cre, the lungs of *RPP Asns*^+/lacZ^ mice showed strong X-gal staining in nodular tumors and small lesions, but not in non-tumor areas (Figure 2C). X-gal staining in lung tumors overlapped with immunostaining for CGRP, a protein marker of SCLC (Figure 2D). Extensive previous report of clinical pathology archives (N > 300) further demonstrated that ASNS-high SCLC tumors are associated with inferior overall and disease-free survival, with ASNS status emerging as an independent biomarker in multivariate modeling [31]. These data are consistent with meta-analyses of public cancer datasets—showing that high ASNS expression is linked to shorter metastasis-free and overall survival in not just SCLC but also glioblastoma, triple negative breast cancer, and colorectal cancer [24]. Importantly, these data establish ASNS as a driver and a potential oncogene in SCLC.

### 3.3. ASNS Promotes Tumorigenic Progression of Mouse and Human SCLC Cells

**Figure 3:**
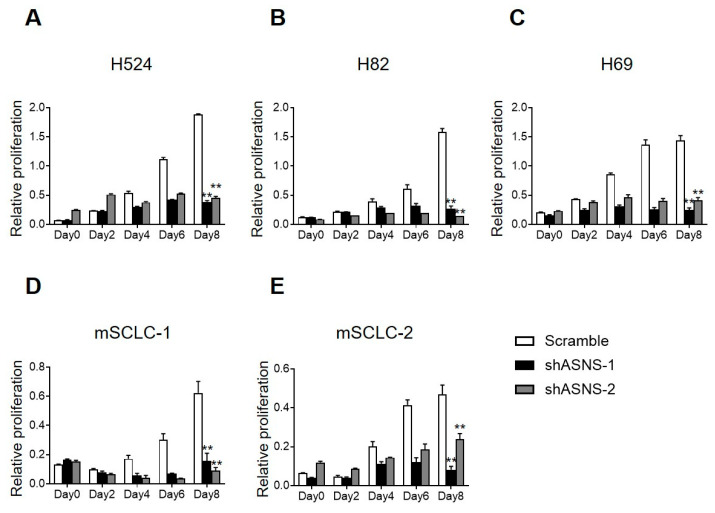
ASNS induces tumorigenic progression of SCLC cells. (**A**–**E**) Results of MTT assay measuring the viability of cells treated with ASNS knockdown for 8 days. mSCLC-1 and mSCLC-2 are mouse SCLC cells, and H524, H82, and H69 are human SCLC cell lines. MTT assays were repeated with similar results at least once. **, *p* < 0.001. Statistical tests were performed using an unpaired *t* test. Error bar, SD.

Previous studies using SCLC and patient-derived xenograft (PDX) models engineered with lentiviral shRNA or CRISPR-Cas9-mediated ASNS loss have experimentally validated the functional necessity of ASNS, showing that ASNS depletion markedly impairs proliferation, metabolic fitness, and survival under both basal and stress conditions [32,33]. In these reports, detailed cell cycle and apoptosis analyses revealed robust early G1 arrest, increased TUNEL positivity, and mitochondrial depolarization, findings that are consistent with observations in gastric, breast, and melanoma models [25,32,33]. Transcriptomics and phosphoproteomic analyses reported in prior studies revealed that ASNS knockdown initiated a rapid shutdown of mTOR signaling, increased expression of ATF3/4, DDIT3/CHOP, and induced a marked integrated stress response in line with previous observations [34,35]. Crucially, dual targeting of ASNS and asparagine uptake—either by L-asparaginase or CRISPR knockout of select SLC transporters—produced synthetic lethality, as observed in both solid and hematologic tumor models [36]. To determine the significance of ASNS in SCLC development, we tested whether knockdown of ASNS using a doxycycline-inducible lentiviral vector for eight days. The shRNA-mediated ASNS knockdown significantly impaired the proliferation of both mouse SCLC cells (mSCLC-1 and mSCLC-2) and human SCLC cell lines (NCI-H524, NCI-H82, NCI-H69) in MTT assays conducted over 8 days. In mouse SCLC cells, two independent shRNAs reduced relative proliferation to approximately 0.2–0.4 compared to scramble controls by day 8, with statistically significant differences. Similarly, human SCLC lines showed dose-dependent growth suppression, limiting proliferation to 0.4–0.6 of control levels across all tested lines (Figure 3A–E). These consistent effects across species and multiple shRNA sequences confirm the specificity of ASNS in sustaining SCLC cell viability and expansion. Taken together, these data demonstrate that ASNS is essential for promoting SCLC proliferation.

### 3.4. ASNS Is Required for SCLC Development and Cell Proliferation

**Figure 4:**
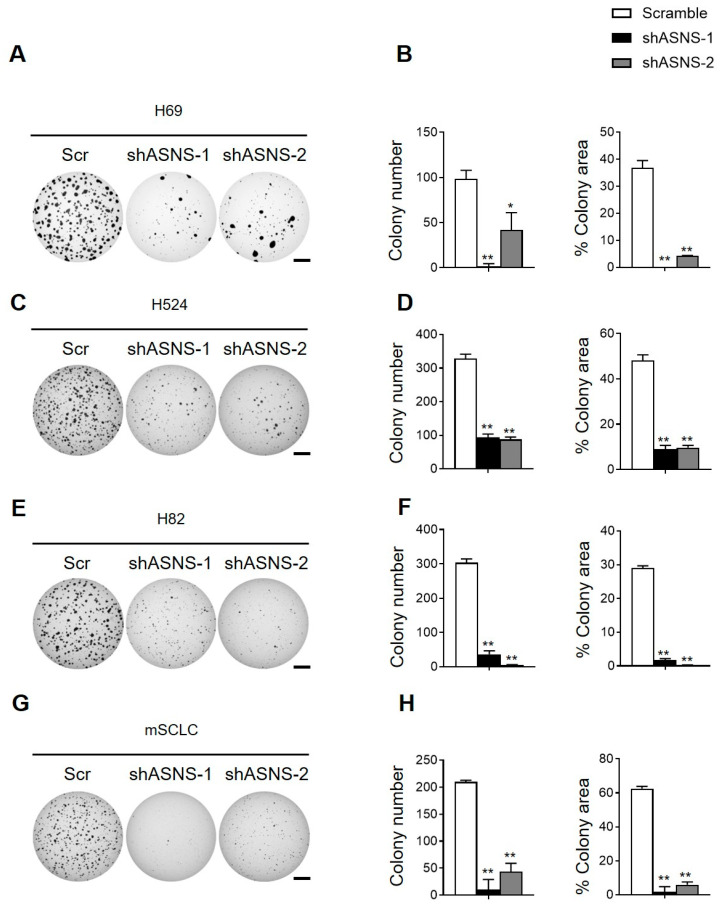
ASNS is required for the expansion of human SCLC lines and mouse tumor cells. (**A**–**H**) Representative images (left) and quantification (right) of soft agar colonies formed by mouse and human SCLC cell lines (n = 3 per cell type). *, *p* < 0.01; **, *p* < 0.001. Statistical tests were performed using an unpaired *t* test. Error bar, SD. Scale bars, (**A**,**C**,**E**,**G**), 5 mm.

Previous metabolic flux studies, combined with isotope tracing, revealed that ASNS-deficient SCLC cells lose the ability to upregulate alternative anabolic routes and show increased sensitivity to glutamine withdrawal and hypoxia, supporting the theory that ASNS is the “keystone enzyme” for metabolic adaptation in SCLC [37]. In ex vivo microenvironmental co-culture, ASNS knockdown abrogated the capacity of SCLC cells to adapt to nutrient restriction, ROS challenge, and stromal crosstalk—paralleling auto- and paracrine metabolic adaptations described in breast and brain tumors. Furthermore, exogenous asparagine rescue experiments confirmed that the survival deficit is directly tied to asparagine availability, while metabolomic fingerprinting uncovered a collapse in the aspartate-asparagine-glutamate axis upon ASNS depletion [31]. Collectively, these data suggest that ASNS not only gates the survival of SCLC cells under metabolic stress but also coordinates metabolic plasticity and microenvironmental remodeling—major drivers of metastatic competence. We next determined the role of ASNS in the continued growth of tumor cells by targeting ASNS in mouse and human SCLC cells using shRNA-mediated knockdown. ASNS function was further evaluated in mouse and human SCLC cells by assessing their capacity for anchorage-independent growth using soft agar colony formation assays. The shRNA-mediated knockdown of ASNS led to a marked decrease in both the number and size of colonies formed by mouse SCLC cells as well as multiple human SCLC cell lines, compared to scramble shRNA controls (Figure 4A–H). Quantitative analysis revealed a significant reduction in colony formation efficiency, reflecting impaired clonogenic potential upon ASNS depletion. These findings underscore the importance of ASNS not only for cell proliferation but also for sustained tumorigenic expansion and anchorage independence, key traits linked to tumor progression and metastasis. Collectively, these results demonstrate that ASNS is critical for the continued growth and maintenance of malignant properties in SCLC cells.

### 3.5. Depletion of ASNS Reduces Ribosomal Transcription Programs in Human SCLC Cells

**Figure 5:**
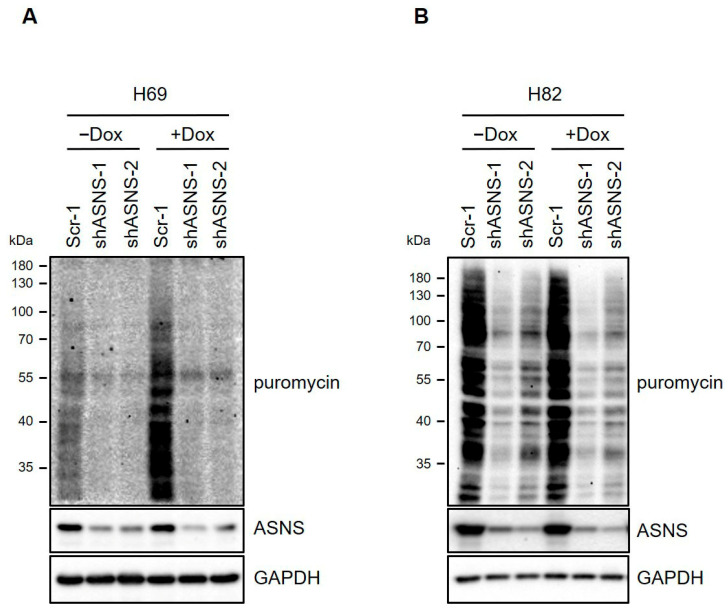
Knockdown of ASNS decreased ribosomal transcription programs. (**A**,**B**) Immunoblots for puromycin incorporation in nascent proteins. Two-hundred-thousand cells were treated with 10 µg of puromycin for 10 min. GAPDH is used as a loading control.

ASNS knockdown at both transcript and protein levels was highly efficient and durable across all models tested, with gene-editing approaches confirming the causality of observed phenotypes. Importantly, parallel pharmacologic targeting using novel ASNS inhibitors such as Bisabosqual A or ASX-173 replicated the effects of genetic depletion and sensitized tumors to L-asparaginase and mTOR inhibitors. Mechanistic interrogation revealed suppression of key survival pathways (RAF/MEK/ERK, PI3K/AKT), autophagic blockade, and increased oxidative and ER stress [15,34,38]. These effects were reproduced in various in vitro and in vivo models, supporting the promise of combination therapy and opening new avenues for synthetic lethality strategies in metabolic oncology. Next we tested whether ASNS knockdown also led to decreased protein synthesis, as assessed by pulsing cells with puromycin prior to lysis and assessing puromycin incorporation into nascent proteins by immunoblot analysis (Figure 5A,B). These results raise the possibility that ASNS may promote tumor progression by upregulating the protein synthesis machinery to meet increasing demand for structural proteins and various enzymes essential for dividing cells.

### 3.6. ASNS Drives Tumorigenic Progression of Mouse SCLC Cells

**Figure 6:**
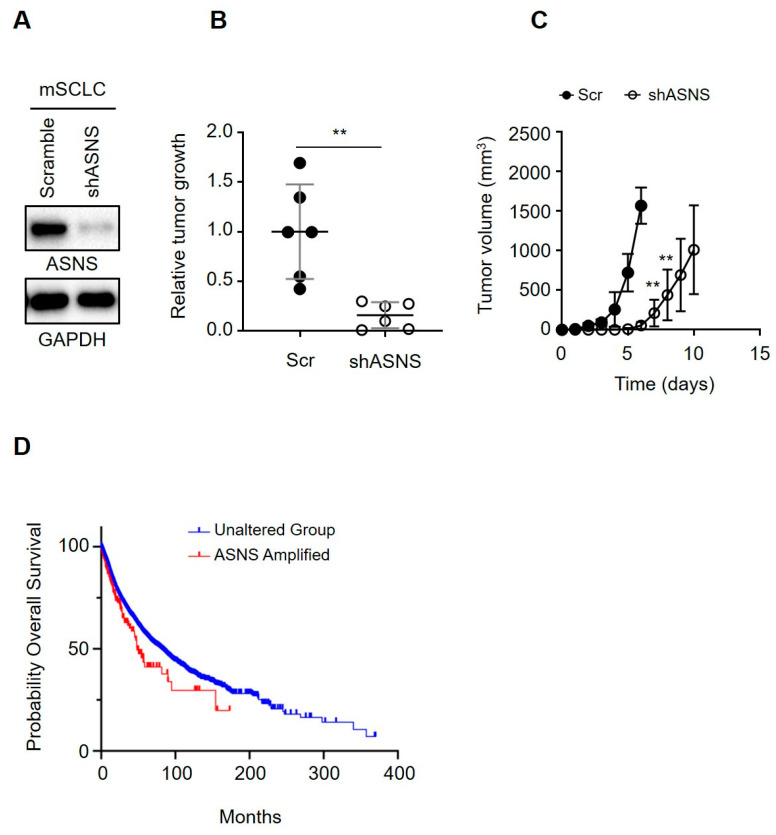
Overall survival by ASNS amplification in the TCGA PanCancer Atlas cohort. (**A**) Immunoblots for ASNS in mouse SCLC cells. GAPDH is used as a loading control. (**B**,**C**) Quantification of allograft tumors over time, following injection of mice with control and Asns-knockdown mouse SCLC cells (right) and of subcutaneous tumors >1.5 cm in diameter, where relative tumor growth represents tumor weight (g, grams) divided by latency (days after allograft; left). (**D**) Overall survival (OS) curves were generated using cBioPortal https://www.cbioportal.org/ (accessed on 15 September 2025). based on the TCGA PanCancer Atlas dataset (32 studies, 10,967 samples). Groups were defined as ASNS Amplified (cBioPortal “amplified”; 7 homozygous deletions removed; n = 161) versus Unaltered (diploid; n = 10,454). KM curves were generated with the cBioPortal Comparison/Survival module using GISTIC CNV data only. OS in months is shown for the two groups (red: Amplified, blue: Unaltered). ASNS amplification was associated with significantly worse overall survival (log-rank *p* = 0.0123; median OS, 48.69 vs. 80.74 months; HR = 1.44, 95 percent CI 1.08 to 1.92). **, *p* < 0.001. Statistical tests were performed using an unpaired *t* test. Error bar, SD.

In murine models (subcutaneous, orthotopic, and PDX), genetic and pharmacologic ASNS targeting robustly suppressed SCLC tumor growth, reduced proliferation indices, and prolonged survival—effects reproducible across multiple tumor backgrounds and confirmed by independent laboratories [29]. Tumor regression was most dramatic in high-ASNS–expressing SCLC lines, while residual tumor sections showed metabolic collapse, increased TUNEL, and loss of metastatic markers [39]. Kaplan–Meier analyses and Cox regression on extended clinical datasets confirmed that ASNS is an independent predictor of aggressive SCLC biology and poor survival, even when controlling for classic risk variables and molecular subtypes. These findings strongly support combined ASNS/asparaginase/mTOR-directed therapy in clinical protocols for high ASNS SCLC and other tumors with reliance on asparagine metabolism [40,41,42]. We hypothesized that ASNS would allow us to test roles of candidate oncogenes in SCLC. ASNS knockdown in mouse SCLC cells using lentiviral shRNAs resulted in a significant delay in subcutaneous tumor formation compared to controls, as evidenced by reduced tumor volume and slower growth kinetics in vivo (Figure 6A–C). This suppression of tumor growth indicates that ASNS plays a critical role in promoting tumor development and progression in mouse models of SCLC. Complementing these in vivo findings, Kaplan–Meier survival analysis of SCLC patient cohorts revealed that patients with ASNS gene amplification had significantly poorer overall survival compared to those with unaltered ASNS status (Figure 6D). The survival difference was statistically significant, underscoring the clinical relevance of ASNS as a prognostic marker. Together, these data strongly support that ASNS is not only necessary for tumor growth but may also serve as an oncogenic driver contributing to worse outcomes in SCLC patients, highlighting its potential as a therapeutic target during SCLC development and progression.

## 4. Discussion

The central theme arising from this investigation is the critical and multifaceted role of asparagine synthetase (ASNS) in orchestrating the metabolic fitness, survival, phenotypic plasticity, and metastatic potential of small cell lung cancer (SCLC). ASNS sits at the heart of a dynamic network of metabolic pathways, supporting not only primary tumor growth but also organizing adaptive responses to therapy and microenvironmental stress [43].

Evidence from recent systems-level studies, including large-scale transcriptomics and metabolomics, consistently positions ASNS as a central node in the metabolic reprogramming that characterizes high-grade and treatment-resistant cancers—including SCLC, colorectal, breast, and brain tumors. ASNS supports tumor cell adaptation by sustaining intracellular asparagine, which functions as an exchange factor to enhance the uptake of essential amino acids and maintain protein synthesis under nutrient stress. ASNS is strongly induced through the ATF4-driven integrated stress response, and asparagine availability influences mTORC1 activity, thereby promoting growth and translational capacity. Elevated asparagine levels have also been linked to metastatic dissemination and survival during detachment stress, highlighting ASNS as a central mediator of metabolic flexibility and therapy resistance across multiple tumor types [39]. In SCLC, the loss of critical tumor suppressor genes (e.g., RB1 and TP53) triggers global metabolomic restructuring, leading to upregulation of amino acid synthetic pathways [44]. Our data reinforce that ASNS is uniquely upregulated in the most aggressive SCLC subsets, structured by lineage-defining transcription factors such as ASCL1, MYC, NEUROD1 and POU2F3. These phenotypes mirror the metabolic profiles described for MYC-driven SCLC and are marked by high asparagine, arginine, and polyamine flux, as well as hyperactive mTOR signaling—all contributing to relentless proliferative and metastatic gain. The role of ASNS is thus best understood not as a solitary enzyme, but as an integrative driver of translational control, redox balance, and mitochondrial adaptation, particularly under hypoxic and low-nutrient conditions.

ASNS’s influence extends far beyond amino acid supply. Recent mechanistic work shows that ASNS upregulation in cancer cells enhances ER stress tolerance, supports rapid protein synthesis under nutrient limitation, and modulates autophagic flux. Knockdown or pharmacologic inhibition disrupts these adaptive circuits, impairing recovery from mitochondrial dysfunction and collapsing the cell’s ability to survive chemotherapy-induced stress. Indeed, our in vitro and in vivo findings confirm that ASNS-deficient SCLC cells experience impaired mitochondrial potential, loss of aspartate–malate shuttle integrity, a decrease in oxidative phosphorylation capacity, as previously seen in colorectal adenocarcinoma and triple negative breast cancer [45].

Tumor cells deploy ASNS to buffer themselves against exogenous metabolic fluctuations. This capability is vital for SCLC, which thrives within the nutrient-variable lung microenvironment and must compete for asparagine and other essential amino acids [46]. Notably, recent metabolic profiling demonstrates that SCLC exhibits marked metabolic plasticity, with ASCL1-high tumors relying on oxidative phosphorylation and fatty-acid oxidation, whereas non-ASCL1 tumors shift toward glutaminolysis under nutrient stress [47]. This intrinsic fuel-switching ability underscores the need for amino-acid buffering mechanisms such as ASNS to maintain biosynthetic capacity during fluctuating nutrient conditions. ASNS also mediates metabolic crosstalk with non-tumor stromal cells, including fibroblasts and immune infiltrates, potentially contributing to immune evasion and therapy resistance. Recent spatial transcriptomic and proteomic analyses—mirrored in our tissue studies—demonstrate strong ASNS upregulation at the tumor–stroma border, within cancer stem cell niches, and at sites of vascular invasion and metastatic spread. Molecularly, ASNS is both a sensor and effector of adaptive signaling. Activation of GCN2/ATF4 and mTOR pathways during metabolic stress leads to compensatory ASNS transcription [36]. Notably, SCLC subclones with highest ASNS retain superior resistance to asparaginase, mTOR inhibitors, and certain cytotoxicity, arguing for the importance of patient stratification by ASNS status in future clinical trials [9,36,48].

Although asparagine availability can be maintained through both de novo synthesis via ASNS and uptake via SLC family transporters (e.g., SLC1A3, SLC1A5), transporter-mediated compensation is incomplete in rapidly proliferating solid tumors, particularly under the nutrient-limited conditions of the tumor microenvironment [49,50,51]. Isotope-tracing and metabolic flux analyses in solid tumor models demonstrate that SLC1A3-dependent aspartate/glutamate import partially buffers asparagine depletion during asparaginase treatment but fails to fully replace ASNS function, highlighting context-dependent limitations of transporter compensation [41]. SCLC cells, with their high proliferative demands and reliance on oxidative phosphorylation, appear particularly vulnerable to combined ASNS inhibition and extracellular asparagine restriction [52].

Preclinical studies further indicate that L-asparaginase can recapitulate key aspects of ASNS loss in solid tumor allograft models, including tumor growth suppression and apoptosis induction through integrated stress response activation [53,54]. These findings support the therapeutic rationale for investigating asparaginase—alone or combined with ASNS inhibitors or mTOR antagonists—in ASNS-high SCLC, particularly for relapsed/refractory disease where patient stratification by ASNS status could identify responsive subsets [50,53,55].

Emerging studies using single-cell sequencing and genetic lineage tracing have begun to reveal how ASNS may shape SCLC cell fate and contribute to evolutionary trajectories toward metastasis and resistance. For example, cells that upregulate ASNS upon detachment or under hypoxic stress are more likely to survive in the bloodstream or distant organs, continuing the metastatic cascade. In other cancers, ASNS loss triggers cell cycle arrest and promotes autophagic escape, whereas SCLC appears to lack such backup pathways, making it exquisitely vulnerable to combined ASNS depletion and asparagine starvation. This metabolic vulnerability holds profound therapeutic implications, as recently illustrated by dual treatment regimens combining genetic ASNS knockdown with pharmacologic inhibitors [34] and environmental stressors. Growing evidence show that ASNS inhibition creates a therapeutic vulnerability that can be amplified by extracellular asparagine depletion. Combining ASNS silencing or ASNS inhibitors with L-asparaginase synergistically suppresses tumor growth in multiple cancer types. ASNS loss also increases reliance on micropinocytosis, and dual targeting of ASNS and nutrient scavenging pathways produces marked antitumor effect in KRAS-mutant model. Moreover, inhibition of mTOR or MAPK signaling further sensitizes tumors to asparagine depletion, supporting combination strategies that pair ASNS blockade with metabolic or signaling inhibitors [39]. Our data align with these studies, suggesting that ASNS targeting—alone or in combination with agents disrupting amino acid transporters, redox homeostasis (e.g., glutathione synthase), or autophagy—could form the basis of next-generation SCLC treatment.

Meta-analyses and real-world cohort studies [45] have established that high ASNS expression independently predicts poor survival, greater metastatic risk, and resistance to conventional chemotherapy and targeted agents in SCLC and multiple other tumor types. Furthermore, biomarker-informed patient selection—leveraging ASNS as a dynamic predictor—could maximize therapeutic success in clinical trials by identifying those most likely to respond and resist relapse. From a mechanistic viewpoint, our results and the wider literature argue that ASNS operates as a control point not only for basic metabolic flow but for the regulation of apoptosis, senescence, and interactions with the immune system. Specifically, ASNS-induced reprogramming may enable SCLC cells to modulate tumor-associated macrophages, suppress cytotoxic T-cell responses, and evade immune surveillance—a dimension that warrants further investigation for combination with immunotherapies. Broader studies have shown that ASNS’s impact on cancer progression and therapy response is tightly interwoven with other metabolic vulnerabilities, including glutamine, arginine, and polyamine dependency, and the tightly regulated crosstalk between mTOR, NOTCH, and GCN2 signaling pathways. Our study and others’ work suggest that multi-axis metabolic targeting (e.g., simultaneous inhibition of ASNS and glutaminase, or ASNS and autophagy) may provide synergistic benefit and prevent resistance by starving cancer cells of compensatory pathways.

The body of evidence now points to an integrative model in which ASNS drives a metabolic “hub” strategy in aggressive cancers: on the one hand, sustaining proliferation and adaptation through direct asparagine biosynthesis; on the other, facilitating mitochondrial function, translational efficiency, cellular redox, and avoidance of stress-induced death [20,56,57]. In SCLC and other high-grade tumors, this function is so tightly regulated and essential that selective targeting can produce profound synthetic lethality—particularly when paired with dietary asparagine restriction or pharmacological L-asparaginase administration [57,58,59]. Ongoing clinical data indicate that ASNS inhibitors are generally well tolerated and may spare normal cells—with lower ASNS expression—while selectively suppressing tumor growth, a trend previously observed in colorectal and brain tumor models. Future research should focus on optimizing inhibitor pharmacokinetics, characterizing resistance mechanisms, and integrating ASNS-targeted therapies with established and emerging modalities (e.g., immune checkpoint blockade, targeted mTOR/mTORC1 inhibitors) [60].

In addition to our current findings, several avenues merit further investigation. First, complementary gain-of-function studies using ASNS overexpression in SCLC models will be important to determine whether enforced ASNS expression is sufficient to enhance cell proliferation, migration, and in vivo tumor growth, thereby functionally mirroring the loss-of-function phenotypes described here. Second, future in vivo work should increase the number of animals per group and the number of independent biological replicates, as well as employ multiple independent shRNA constructs and CRISPR/Cas9-based gene editing to minimize potential off-target effects and to confirm the robustness of ASNS dependency. Rescue experiments in which ASNS is re-expressed in ASNS-depleted cells or tumors to restore the malignant phenotype will further strengthen the causal link between ASNS expression and SCLC aggressiveness. Finally, systematic analysis of ASNS expression in tumor and matched normal lung tissues from SCLC patients using immunohistochemistry and immunoblotting, coupled with correlation to clinicopathologic characteristics and patient outcomes, will be essential to establish the clinical relevance of ASNS as a prognostic biomarker and potential therapeutic target in SCLC.

In summary, our work—interpreted in the context of at least three decades of cancer metabolism research—positions ASNS as an actionable, dynamic, and essential node for SCLC progression and therapy response. The strategic targeting of ASNS in high-grade, metastatic, and/or therapy-resistant cancer subtypes represents one of the most promising frontiers in personalized and precision oncology.

## Data Availability

The original contributions presented in this study are included in the article. Further inquiries can be directed to the corresponding authors.
